# Toxicity of Carbon, Silicon, and Metal-Based Nanoparticles to Sea Urchin *Strongylocentrotus intermedius*

**DOI:** 10.3390/nano10091825

**Published:** 2020-09-13

**Authors:** Konstantin Pikula, Alexander Zakharenko, Vladimir Chaika, Iurii Em, Anna Nikitina, Evgenii Avtomonov, Anna Tregubenko, Alexander Agoshkov, Ilya Mishakov, Vladimir Kuznetsov, Alexander Gusev, Soojin Park, Kirill Golokhvast

**Affiliations:** 1 Far Eastern Federal University, Sukhanova 8, 690950 Vladivostok, Russia; zakharenko.am@dvfu.ru (A.Z.); chayka.vv@dvfu.ru (V.C.); em.ium@dvfu.ru (I.E.); nikitina.av@dvfu.ru (A.N.); avtomonov.eg@dvfu.ru (E.A.); tregubenko.ayu@dvfu.ru (A.T.); agoshkov.ai@dvfu.ru (A.A.); golokhvast.ks@dvfu.ru (K.G.); 2 N.I. Vavilov All-Russian Institute of Plant Genetic Resources, B.Morskaya 42-44, 190000 Saint-Petersburg, Russia; 3 Boreskov Institute of Catalysis SB RAS, Lavrentiev Ave. 5, 630090 Novosibirsk, Russia; mishakov@catalysis.ru (I.M.); kuznet@catalysis.ru (V.K.); 4 Tambov State University Named after G.R. Derzhavin, Internatsionalnaya 33, 392000 Tambov, Russia; nanosecurity@mail.ru; 5 National University of Science and Technology «MISIS», Leninskiy prospekt 4, 119049 Moscow, Russia; 6 Inha University, 100 Inharo, Nam-gu, Incheon 22212, Korea; sjpark@inha.ac.kr; 7 Pacific Geographical Institute, Far Eastern Branch of the Russian Academy of Sciences, Radio 7, 690041 Vladivostok, Russia

**Keywords:** aquatic toxicology, bioassay, ecotoxicology, embryo, metal nanoparticles, nanofibers, nanotubes, nanotoxicology, sea urchin development

## Abstract

With the increasing annual production of nanoparticles (NPs), the risks of their harmful influence on the environment and human health are rising. However, our knowledge about the mechanisms of interaction between NPs and living organisms is limited. Prior studies have shown that echinoderms, and especially sea urchins, represent one of the most suitable models for risk assessment in environmental nanotoxicology. To the best of the authors’ knowledge, the sea urchin *Strongylocentrotus intermedius* has not been used for testing the toxicity of NPs. The present study was designed to determine the effect of 10 types of common NPs on spermatozoa activity, egg fertilization, and early stage of embryo development of the sea urchin *S. intermedius*. In this research, we used two types of multiwalled carbon nanotubes (CNT-1 and CNT-2), two types of carbon nanofibers (CNF-1 and CNF-2), two types of silicon nanotubes (SNT-1 and SNT-2), nanocrystals of cadmium and zinc sulfides (CdS and ZnS), gold NPs (Au), and titanium dioxide NPs (TiO_2_). The results of the embryotoxicity test showed the following trend in the toxicity level of used NPs: Au > SNT-2 > SNT-1 > CdS > ZnS > CNF-2 > CNF-1 > TiO_2_ > CNT-1 > CNT-2. This research confirmed that the sea urchin *S. intermedius* can be considered as a sensitive and stable test model in marine nanotoxicology.

## 1. Introduction

The growing industry of nanotechnology has generated reasonable concerns regarding the issues of safety and risk assessment for nanoparticles (NPs) [1]. Different types of NPs are widely applied in a variety of industrial and consumer products [2]. Carbon nanotubes and nanofibers are one of the most promising classes of nanomaterials, widely used in electronics [3], catalysis [4], environmental purification [5], biomedicine [6], energy storage [7], and other fields. Silica nanotubes are used in drug delivery [8], catalysis [9], and biosensing [10] due to their mesoporous properties [11]. Metal sulfide NPs, including cadmium and zinc sulfides (CdS, ZnS) are often used for the oxidation of organic matter during sewage water treatment due to their photocatalytic properties [12,13]. Titanium dioxide (TiO_2_) NPs are used in a variety of consumer products and are also widely applied in environmental purification [14]. Gold (Au) NPs have promising applications in medicine, biology, and chemistry [15]. Moreover, it was reported that NPs can enter the environment during their manufacturing, utilization, and disposal [16]. A large and growing body of literature has investigated the aquatic toxicity of carbon nanotubes [17,18], carbon nanofibers [19], silicon NPs [20], and metal-based NPs [21,22] in different model organisms. However, it is still difficult to understand the mechanisms of toxicity and behavior of different types of nanomaterials in aquatic systems.

The reproductive, developmental, and embryotoxicity of NPs represent a particular area of interest to scientists in the area of nanotoxicology [23,24,25,26]. Prior studies have shown that echinoderms, and especially sea urchins, represent one of the most suitable models in the assessment of reproductive, developmental, and embryotic toxicity of NPs in the aquatic environment [27,28,29]. Sea urchins have been described as a key species of marine benthic communities attached to hard bottoms, with habitats ranging from a few centimeters deep to 20 or more meters [30]. Sea urchin, and general echinoderm, fertilization takes place outside the maternal body [27]. Embryos and larvae of sea urchins can swim in the water column and represent good food for other organisms [27].

In this work, we used the sea urchin *Strongylocentrotus intermedius*. The sea urchin *S. intermedius* can be found in the Northwest Pacific region, including the Sea of Japan and off the coast of the Korean Peninsula [31,32]. To the best of the authors’ knowledge, the sea urchin *S. intermedius* has not been used for testing the toxicity of NPs. The current study demonstrated the sensitivity and stability of *S. intermedius* as a model organism and demonstrated the difference in the toxic effects caused by the influence of 10 types of common NPs i.e., two types of multiwalled carbon nanotubes (CNT-1, CNT-2), two types of carbon nanofibers (CNF-1, CNF-2), two types of silicon nanotubes (SNT-1, SNT-2), nanocrystals of cadmium and zinc sulfides (CdS, ZnS), Au NPs, and TiO_2_ NPs on spermatozoa activity, egg fertilization, and early stage of embryo development of the sea urchin *S. intermedius*. The comparison of the toxic effects caused by the different types of carbon and silicon NPs, and the dependence of these effects on the physicochemical properties and structural features of the NPs represents a significant area of research interest. The toxicity evaluation of the NPs with relatively low toxicity, such as TiO_2_ [33] and Au [34], in new test objects with an application of different types of bioassays, is an important part of NP risk assessment.

## 2. Materials and Methods

### 2.1. Nanoparticles

Characteristics of all the NPs used in this study are represented in Table 1.

Carbon nanotubes and nanofibers were synthesized and characterized in the Boreskov Institute of Catalysis (Novosibirsk, Russia) [35]. Silicon nanotubes were kindly provided by the Department of Chemistry, Inha University Republic of Korea [36]. CdS and ZnS NPs were synthesized and characterized in the University of Mining and Geology, St. Ivan Rilski (Sofia, Bulgaria) [38]. The sample Au was synthesized by the Turkevich method with a reduction by citrate at 100 °C [39]. TiO_2_ nanopowder was purchased from Thermo Fisher Scientific (Thermo Fisher GmbH, Kandel, Germany, CAS number 1317-70-0, product number 39953).

### 2.2. Sea Urchin Toxicity Bioassay

**Figure 1:**
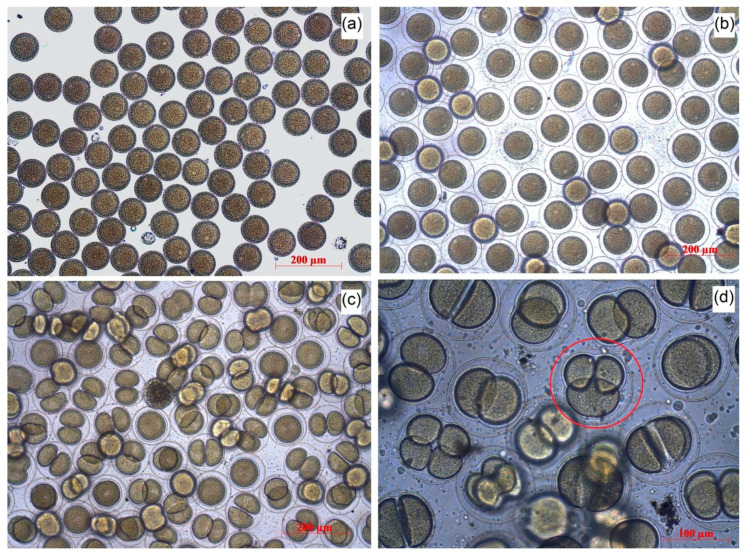
The stages of *S. intermedius* embryo development: (**a**) unfertilized eggs; (**b**) fertilized eggs; (**c**) normal embryo development; (**d**) the example of impaired development.

Adult specimens of the sea urchin *Strongylocentrotus intermedius* (Agassiz, 1864) were collected from Novi Dzhigit bay (Peter the Great Bay, Sea of Japan, Russia). Male and female gametes were received and fertilized according to the modified method of Buznikov [40]. The sterile seawater used in the experiment was prepared by filtering fresh seawater (filter pore 0.22 μm) and heating in a microwave for 10 min. The obtained gametes were washed and diluted with the prepared water. The quality of the obtained material was controlled by the trial fertilization of the eggs. More than 99.8% of the cells (Figure 1a) formed the fertilization membrane (Figure 1b), which confirmed that the obtained material was suitable for bioassays.

To prepare the stock suspension of the NPs for the bioassays, the NPs were added to the prepared sterile seawater to obtain a concentration of 1000 mg/L. Before each series of bioassays, the working suspensions of NPs were sonicated with an ultrasound homogenizer Bandelin Sonopuls GM 3100 (Bandelin Electronic GmbH & Co. KG, Berlin, Germany).

To assess the toxicity of the NPs, we performed three types of tests, namely (1) a spermiotoxicity test, (2) an egg fertilization inhibition, and (3) an early-stage embryo development test. The incubation was carried out in 24-well plates at a temperature of 18–20 °C. All of the experiments were performed in quadruplicate. The cell aliquot in each replication had a volume of 1000 μL. The NPs were added to 900 μL of urchin cells in the volume of 100 μL to obtain the final concentrations of 0.1, 1, 10, 25, 50, and 100 mg/L. Then, 100 μL of sterile seawater was added to the control groups. For each replication, at least 100 cells were observed by an inverted optical microscope Axio Observer Z1 (Carl Zeiss, Oberkochen, Germany).

The inhibition of spermatozoa activity was measured by a 30 min exposition of spermatozoa aliquot (1 × 10^7^ cells/mL) to the NPs. The motility of the spermatozoa was observed by microscopy. Then, 100 μL of unfertilized eggs (2 × 10^4^ cells/mL) were added to the exposed spermatozoa.

The inhibition of egg fertilization was measured by a 30 min exposition of unfertilized eggs of *S. intermedius* (2 × 10^3^ cells/mL) to the NPs while adding a further 100 μL of the spermatozoa (1 × 10^8^ cells/mL). Then, the number of fertilized eggs was counted in relation to the total number of eggs.

In the embryo development test, the NPs were added to the eggs of *S. intermedius* 5 min after the fertilization. The fertilization was performed by adding 100 μL of spermatozoa (1 × 10^8^ cells/mL) to 800 μL of unfertilized eggs (2 × 10^3^ cells/mL). The observation was carried out for 48 h with control measurements after 2, 4, 6, 24, and 48 h of the NPs exposition. The embryos that reached correct development in each endpoint were defined as normal. The normal development includes the following features: (1) the mid gastrula formed at 24 h, (2) the cells possessed left/right and dorso/ventral body symmetry, (3) the cells showed well-differentiated archenteron at 24 h, (4) the cells exhibited fully developed arms and complete skeletal rods at the pluteus stage. The number of normally developed embryos (Figure 1c), the embryos with developmental delays or impaired development (Figure 1d), and dead embryos were counted in relation to the total number of zygotes.

### 2.3. Statistical Analysis

Statistical analyses were performed using the software package GraphPad Prism 8.0.2 (GraphPad Software, San Diego, CA, USA). The one-way ANOVA test was used for analysis. A value of *p* ≤ 0.05 was considered statistically significant.

## 3. Results

The spermiotoxicity test revealed no inhibition of spermatozoa activity of the sea urchin *S. intermedius* at the used concentrations for all of the tested NPs, except for the samples of SNT-2 and Au. The samples SNT-2 and Au caused a pronounced inhibition of spermatozoa motility after the exposition to the concentrations of 50 and 100 mg/L.

**Figure 2:**
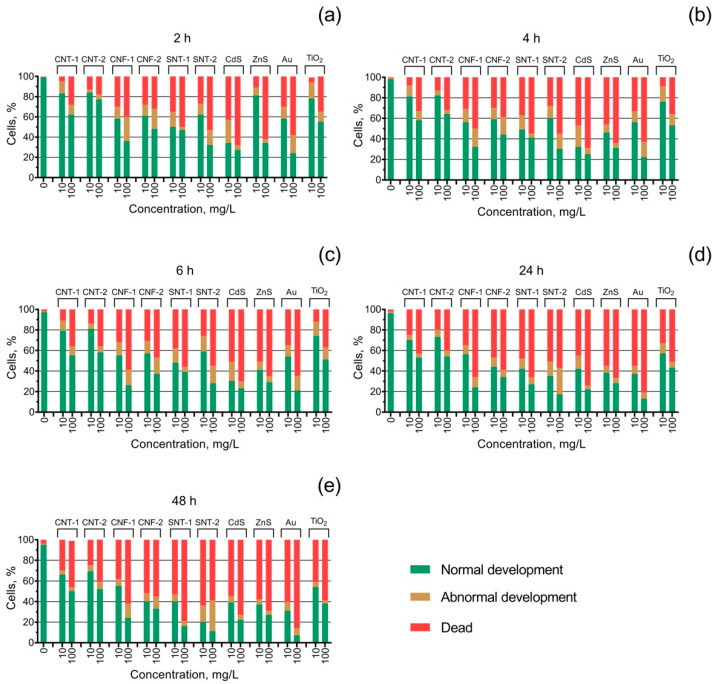
The state of *S. intermedius* embryo development under the exposure to the tested nanoparticles at the concentrations of 10 and 100 mg/L: (**a**) 2 h of the exposition; (**b**) 4 h of the exposition; (**c**) 6 h of the exposition; (**d**) 24 h of the exposition; (**e**) 48 h of the exposition.

The inhibition of egg fertilization and embryo mortality of the sea urchin *S. intermedius* under the influence of the tested NPs is represented in Table 2 as the calculated values of EC_50_ (the concentrations of the NPs that caused 50% inhibition of sea urchin egg fertilization) and LC_50_ (the concentrations of the NPs that caused 50% mortality of the embryos), respectively. Abnormalities in the embryo development caused by the influence of the NPs were shown in Figure 2. To demonstrate the dynamics of the effect, we have shown only the middle and the highest-used concentrations in the figure, which were 10 and 100 mg/L, respectively.

According to the results of the egg fertilization inhibition test (Table 2), the EC_50_ values lower than 200 mg/L were registered for the tested metal sulfide NPs (ZnS and CdS), for one type of the used silicon NPs (SNT-2), and metal NPs (Au). The highest inhibition of egg fertilization was caused under the influence of the sample ZnS (EC_50_, 119.4 mg/L). The samples TiO_2_ and CNF-1 demonstrated a relatively low level of toxicity in this type of bioassay (EC_50_, 620.6 and 830.4 mg/L, respectively). The lowest inhibition of egg fertilization was registered for both types of carbon nanotubes (CNT-1 and CNT-2), carbon nanofibers CNF-2, and silicon nanotubes SNT-1.

The registered mortality of the embryos of *S. intermedius* under the influence of the tested NPs (Table 2) revealed the highest acute toxicity (2 h of exposure) of the samples CdS, ZnS, Au, and SNT-1 (LC_50_, 26.6, 35.8, 46.5, and 48.1 mg/L, respectively). The lowest acute toxicity in the embryo mortality test was registered for the samples CNT-1, TiO_2_, and CNT-2 (LC_50_, 255.5, 232.0, and 297.1 mg/L, respectively). The embryotoxicity of all the tested NPs was increased with time. It should be noted that the calculated 48 h LC_50_ values were lower or close to 10 mg/L for the samples Au, SNT-2, SNT-1, ZnS, and CdS (LC_50_, 4.3, 6.0, 8.4, 10.6, and 10.6 mg/L, respectively). These results correlate with the results of the inhibition of egg fertilization test, where the same samples of NPs (except SNT-1) demonstrated the highest level of toxicity. The lowest chronic toxicity (48 h of the exposure) in the embryo mortality test was registered for the samples CNT-2 and CNT-1 (LC_50_, 75.5 and 66.7 mg/L, respectively). The chronic embryotoxicity level of the tested NPs had the following trend (from the most toxic to the less toxic sample): Au > SNT-2 > SNT-1 > CdS > ZnS > CNF-2 > CNF-1 > TiO_2_ > CNT-1 > CNT-2.

The early-stage embryo development test (Figure 2) showed an increase in the number of cases with abnormal embryo development by more than 10% for all the tested samples except CNT-1, CNT-2, and ZnS. The sample CdS caused a higher embryo development disorder at a lower concentration (10 mg/L) compared to the highest-used concentration (100 mg/L), and it was reduced with time accompanied by an increase in the level of mortality of the embryos. The highest level of abnormal sea urchin embryo development (30%) was registered for the sample of silicon nanotubes SNT-2 after 48 h of exposure at a concentration of 100 mg/L (Figure 2e). In the case of SNT-2 both the level of embryo mortality and development disorder increased with time.

## 4. Discussion

In this study, the spermiotoxicity test of the sea urchin *S. intermedius* demonstrated low sensitivity to the tested NPs, and spermatozoa activity was inhibited only under the highest-used concentrations (50 and 100 mg/L) of two samples with the highest embryotoxicity (Au and SNT-2). These results are in agreement with previous studies, which found that the sperm fertilization capability of the sea urchin *Paracentrotus lividus* was not affected by the exposure of SiO_2_, SnO_2_, CeO_2_, Fe_3_O_4_, Ag, TiO_2_, and Co NPs, while the toxic effect was dramatic for the embryos [27,41,42]. The higher level of sea urchin embryotoxicity (48 h LC_50_ about 10 mg/L) was observed for metal (Au), metal sulfide (CdS and ZnS), and silicon (SNT-1 and SNT-2) NPs (Table 2). Carbon nanofibers also demonstrated relatively high embryotoxicity (48 h LC_50_ about 25 mg/L), and carbon nanotubes were the least toxic (48 h LC_50_, 65–75 mg/L). These results are consistent with our previous research with the same NPs tested in microalgae [43,44] and bivalves [45], where the highest toxicity was observed for the samples of metal and metal sulfide NPs, followed by silicon nanotubes, carbon nanofibers, and carbon nanotubes.

Recent studies showed that the power of absorption to cell membranes played a considerable role in the level of toxicity of NPs in sea urchin models [42,46]. Previously, we demonstrated in microalgae models the dependence of CdS and ZnS toxicity on cell membrane properties [44]. In this work, the metal and metal sulfide NPs (Au, CdS, and ZnS) had a similar level of embryotoxicity and inhibition of sea urchin egg fertilization (Table 2). The high aquatic toxicity of these NP samples could be associated with their high photoactivity [38,47], which facilitates the generation of reactive oxygen species (ROS) in the media and, finally, leads to the oxidative stress, DNA damage, and general cellular metabolic disorders in sea urchin cells [28,48]. The relatively low embryotoxicity and inhibition of the sea urchin *S. intermedius’* egg fertilization when exposed to TiO_2_ NPs correlates with the previously reported results of the bioassay with the sea urchin *Lytechinus pictus*, where no adverse effects were registered after 96 h of the exposure to the TiO_2_ NPs at concentrations up to 10 mg/L [49]. The other works showed time-dependent clustering of TiO_2_ NPs, suggesting that the level of NP toxicity may not have a direct dose dependence and that the less-concentrated suspensions can cause major effects on the morphology and biochemistry in sea urchin models [42,50]. However, our results (Table 2) revealed a significant time-dependent increase in the level of embryotoxicity of TiO_2_ NPs to the sea urchin *S. intermedius*. The toxic effect was the most profound after 24 and 48 h of exposure (Table 2). A further study should assess the long-term influence of TiO_2_ NPs on marine echinoderms.

To date, relatively few studies have assessed the toxic influence of carbon and silicon NPs on sea urchin models [51,52]. In our study, we can highlight the difference in the level of embryotoxicity between silicon nanotubes (48 h LC_50_ value below 10 mg/L), carbon nanofibers (48 h LC_50_ value 25 mg/L), and carbon nanotubes (48 h LC_50_ value 65–75 mg/L) (Table 2).

An interesting finding was that the sample of silicon nanotubes SNT-1 had a very low effect on sea urchin egg fertilization despite the high embryotoxicity (Table 2). At the same time, the silicon nanotube sample SNT-2 was one of the most toxic tested NPs in all three tests (spermiotoxicity, egg fertilization inhibition, and embryotoxicity). On the other hand, the influence of SNT-2 caused the highest embryo development disorder (Figure 2) compared to the other tested NPs. The high impact of SiO_2_ NPs on the early development stages of the sea urchin *P. lividus* and the high percentage of undeveloped and anomalous embryos has been reported previously [41]. Most likely, the high embryotoxicity could be related to the hydrophilic properties of silica NPs, which prevent the conglomeration of the particles and allow them to move easily in the water body [53]. According to the absence of impurities in the composition of SNT-1 and SNT-2 samples (Table 1), we can conclude that the main toxic mechanism for silica nanotubes was the mechanical damage of the sea urchin cells. However, we cannot highlight the direct dependence of the toxicity level of the tested silica NPs on their size and surface area (Table 1).

In general, the sea urchin *S. intermedius* as a model of the bioassay demonstrated the highest tolerance to the carbon nanotubes CNT-1 and CNT-2 among the other tested samples (Table 2). Between these two samples, the sample CNT-1 had the higher toxic influence, which also had a higher amount of metal impurities and unordered structure (Table 1). The tested carbon nanofibers had a significantly higher influence on the *S. intermedius* compared to the carbon nanotubes, which can be associated with the higher number of defect areas and the more unordered structure of these samples. It was shown that carbon NPs can bind to the membranes of micro-organisms by hydrophobic interaction and hydrogen bonding formed between surfaces of cells and defect areas of NPs [54].

Our research demonstrated that the sea urchin *S. intermedius* can be used in the testing of NPs. However, we believe that further investigations of NP environmental risk assessment should focus on the incorporation of multiomics approaches, predicting-toxicity tools, and the evaluation of the problems of biocorona formation and biotransformation of NPs by the organisms and in the media [55,56,57,58].

## 5. Conclusions

To our knowledge, this is the first study that investigates the effects of NPs on spermatozoa activity, egg fertilization, and early stage of embryo development of the sea urchin *S. intermedius*. This study demonstrated that the chosen model represents a simple, reliable, and sensitive method of NP risk assessment, and the bioassays on *S. intermedius* can be successfully applied in marine nanotoxicology.

The highest embryotoxicity (48 h LC_50_ value 6–10 mg/L) was registered for both samples of silicon nanotubes, CdS, ZnS, and Au NPs. The same metal and metal-based NPs and only the one sample of silicon nanotubes (SNT-2) caused pronounced inhibition of sea urchin egg fertilization (EC_50_ value 132–182 mg/L). The spermiotoxicity test of the sea urchin *S. intermedius* demonstrated low sensitivity to the NPs, and spermatozoa activity was inhibited only under the highest-used concentrations (50 and 100 mg/L) of two samples with the highest embryotoxicity (Au and SNT-2).

Among the most common mechanisms of the reproductive and developmental toxicity of NPs, many authors highlighted oxidative stress, inflammation, and DNA damage. However, much uncertainty still exists about the relationship between multiple important factors affecting the aquatic behavior of NPs, such as particle size, surface area, surface chemistry, crystalline structure, method of synthesis, purity, particle transformation, interaction with organic matter, etc. Therefore, the evaluation of the aquatic toxicity of NPs requires further complex and multiparametric study with a variety of different model-organisms and test endpoints.

## Figures and Tables

**Table 1:** Characteristics of the nanoparticles (NPs) used in the experiment.

Sample	Diameter, nm	Surface Area, m^2^/g	Impurities, %	Structural Features
CNT-1	18–20	130	Al—0.9	Many particles with defect areas and opened ends of carbon nanotubes
			Co—0.3	
			Fe—0.6	
CNT-2	18–20	150	Ca—0.004	Ordered nanotube structure
			Cl—0.08	
			Co—0.12	
			Fe—0.2	
CNF-1	90–120	90–100	Al_2_O_3_—0.4	Unordered structure, defect areas, the presence of amorphous carbon
CNF-2	90–120	90–100	Al_2_O_3_—0.4	Unordered structure, defect areas
			Ni—3.6	
SNT-1	3–4	685	—	Ordered nanotube structure
SNT-2	40–45	395	—	Ordered nanotube structure
Cadmium sulfide (CdS)	5–9	n/a	—	Cubic crystal phase
Zinc sulfide (ZnS)	2.6–5.6	n/a	—	Cubic crystal phase
Gold (Au)	60–80	n/a	—	Spherical shape
Titanium dioxide (TiO_2)_	32	n/a	Total metal—0.1	Nanopowder, anatase crystal structure

The characteristics given in the table are as given in the earlier studies [35,36,37,38].

**Table 2:** The mean calculated EC_50_ values of the inhibition of egg fertilization and the mean calculated LC_50_ values of embryo mortality of the sea urchin *S. intermedius*.

Sample	Egg Fertilization Inhibition ^1^, EC_50_, mg/L	Embryo Mortality, LC_50_, mg/L				
		2 h ^1^	4 h ^1^	6 h ^2^	24 h ^3^	48 h ^3^
CNT-1	>1000	255.5 (187–372)	193.2 (136–293)	163.6 (115–245)	85.5 (36–244)	66.7 (21–263)
CNT-2	>1000	297.1 (152–950)	192.3 (126–325)	165.1 (109–271)	97.0 (43–270)	75.5 (31–218)
CNF-1	830.4 (490–2154)	78.5 (26–323)	54.5 (20–166)	43.7 (18–109)	29.8 (11–79)	25.2 (7–82)
CNF-2	>1000	93.6 (36–318)	77.5 (25–342)	48.9 (16–168)	29.1 (5–134)	24.6 (1–178)
SNT-1	>1000	48.1 (15–165)	42.2 (13–139)	37.9 (12–125)	17.5 (1–111)	8.4 (0.1–65)
SNT-2	144.8 (72–376)	80.2 (36–207)	66.3 (27–187)	58.2 (22–167)	22.5 (1–289)	6.0 (4–130)
CdS	131.9 (44–942)	26.6 (9–69)	22.3 (5–74)	18.3 (2–80)	21.6 (1–117)	10.6 (0.1–92)
ZnS	119.4 (36–1027)	35.8 (5–202)	28.2 (4–144)	22.1 (1–145)	15.7 (1–170)	10.6 (0.1–114)
Au	182.1 (108–362)	46.5 (14–173)	42.5 (12–160)	31.7 (7–122)	15.1 (1–98)	4.3 (0.1–30)
TiO_2_	620.6 (484–845)	232.0 (147–426)	192.9 (123–341)	161.8 (101–288)	51.6 (21–47)	32.3 (6–155)

^1^*p* < 0.0001; ^2^ *p* < 0.001; ^3^ *p* < 0.05; 95% confidence limits presented in the parentheses.
